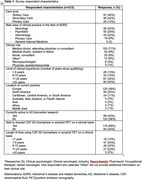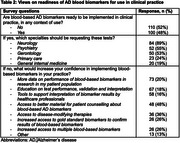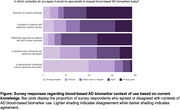# Global multi‐specialty clinician perspectives on the implementation of Alzheimer's disease blood biomarkers

**DOI:** 10.1002/alz70856_100267

**Published:** 2025-12-25

**Authors:** Joanne Rodda, Lindsey A. Kuchenbecker, Wyllians Vendramini Borelli, Mari L DeMarco, Erin E Cawston, Tinatin Chabrashvili, Melissa M Budelier, Claudia Duran‐Aniotz, Chinedu T Udeh‐Momoh, Leyla Anderson, Michelle M Mielke, Ana C Pereira, Alicia Algeciras‐Schimnich, Ashvini Keshavan, Raphael Machado Castilhos

**Affiliations:** ^1^ Kent and Medway NHS and Social Care Partnership Trust, Maidstone, Kent, United Kingdom; ^2^ Kent and Medway Medical School, Canterbury, Kent, United Kingdom; ^3^ Mayo Clinic, Jacksonville, FL, USA; ^4^ Mayo Clinic, Rochester, MN, USA; ^5^ Universidade Federal do Rio Grande do Sul, Porto Alegre, Rio Grande do Sul, Brazil; ^6^ Centro de Memória, Hospital Moinhos de Vento, Porto Alegre, RS, Brazil; ^7^ University of British Columbia, Vancouver, BC, Canada; ^8^ Providence Health Care, Vancouver, BC, Canada; ^9^ Centre for Brain Research, The University of Auckland, Auckland, New Zealand; ^10^ SUNY Upstate Medical University, Syracuse, NY, USA; ^11^ TriCore Reference Laboratories, Albuquerque, NM, USA; ^12^ Latin American Brain Health Institute (BrainLat), Universidad Adolfo Ibañez, Santiago, Chile; ^13^ Wake Forest University School of Medicine, Winston‐Salem, NC, USA; ^14^ Brain and Mind Institute, Aga Khan University, Nairobi, Kenya; ^15^ NeuroVision Imaging, Inc, Sacramento, CA, USA; ^16^ Division of Public Health Sciences, Wake Forest University, School of Medicine, Winston‐Salem, NC, USA; ^17^ Icahn School of Medicine at Mount Sinai, New York, NY, USA; ^18^ Dementia Research Centre, UCL Queen Square Institute of Neurology, University College London, London, United Kingdom

## Abstract

**Background:**

Clinicians’ views on clinical readiness of Alzheimer's disease (AD) blood biomarkers (BBM) are not well understood.

**Method:**

The Alzheimer's Association International Society to Advance Alzheimer's Research and Treatment Biofluid‐Based Biomarkers Professional Interest Area conducted a survey to elicit clinician opinions on AD BBM implementation, including contexts of use, assay selection, reporting and result interpretation.

**Result:**

Clinician respondents (*n* = 212) practiced in Europe (56%), North America (24%), Caribbean and Central or South America (11%) and other continents (9%). Most respondents were medical doctors (80%) practicing in secondary or tertiary care (88%) (Table 1). For 56% of respondents, CSF AD biomarkers or amyloid PET were accessible, but 48% agreed and 52% disagreed with implementation of AD BBM in any clinical context (Table 2). Respondents emphasized the need for data from diverse populations and educational resources to support test interpretation.

**Conclusion:**

Surveyed clinicians generally agreed with published appropriate use recommendations but were divided on AD BBM readiness for clinical use.